# Evaluation of DDGS as a Low-Cost Feed Ingredient for Common Carp (*Cyprinus carpio* Linneus) Cultivated in a Semi-Intensive System

**DOI:** 10.3390/life12101609

**Published:** 2022-10-14

**Authors:** Cristian-Alin Barbacariu, Cristina Mihaela Rimbu, Lenuta Dirvariu, Marian Burducea, Razvan Stefan Boiangiu, Elena Todirascu-Ciornea, Gabriela Dumitru

**Affiliations:** 1 Research and Development Station for Aquaculture and Aquatic Ecology, “Alexandru Ioan Cuza” University, Carol I, 20A, 700505 Iasi, Romania; 2 Department of Public Health, Faculty of Veterinary Medicine, University of Life Sciences ‘’Ion Ionescu de la Brad’’ Iaşi, Mihail Sadoveanu Alley 6-8, 700490 Iasi, Romania; 3 Faculty of Biology, “Alexandru Ioan Cuza” University, Carol I, 20A, 700505 Iasi, Romania

**Keywords:** aquaculture, common carp, diets, alternative ingredient, production, flesh quality, oxidative status, intestinal microflora

## Abstract

Distillers dried grains with solubles (DDGS), a coproduct from the ethanol production industry, is successfully used as an ingredient in feeding cattle and pigs due to its relatively high protein and nutrient content and low price compared to cereals. The aim of this study was to establish the optimal DDGS concentration that can be included in the diet of common carp. A seven-week experiment was performed on common carp with an initial weight of 86 g feed with three experimental diets D0 (DDGS 0%), D1 (DDGS 25%) and D2 (DDGS 35%). The chemical composition of DDGS analyzed by Fourier Transform Near-Infrared (FT-NIR) spectroscopy showed a protein content of 27.56% and oil at 6.75%. Diets with DDGS did not produce significant changes in growth parameters, flesh quality, and blood biochemical profile. Regarding the oxidative status in the muscle tissue, D1 and D2 significantly reduced, in a dose-dependent manner, the specific activity of SOD and GSH, while CAT and GPX were left unaffected. In the liver tissue, CAT, GSH, MDA and carbonylated proteins were reduced in the DDGS diets. The microbiological analysis of the intestinal contents revealed a variation in microbial density depending on the diet used. The total number of aerobic germs was between 224.2 × 10^4^ and 69.84 × 10^6^ (D2 > D1 > D0) and the total number of anaerobic germs was between 15.2 × 10^2^ and 28.2 × 10^2^ (D2 > D0 > D1).

## 1. Introduction

Common carp (*Cyprinus carpio* L.) is one of the most important aquaculture species in Asian and Central and Eastern European countries [1]. According to Eurostat, in 2018, the main European Union producing countries of common carp and other Cyprinidae species were the Czech Republic (19.6 thousand tons), Poland (18 thousand tons), Hungary (13.3 thousand tons), and Romania (9.5 thousand tons) [2]. Common carp are generally cultivated in extensive or semi-intensive systems following traditional methods. In these rearing systems, the feed is represented by the natural live organisms found in water and supplementary added cereals. Cereals are primary processed only, by grinding to reduce the particle size and improve nutrient availability or by soaking to improve palatability and eliminate anti-nutritional factors [3]. Due to the high initial investment costs, grain extrusion and granulation is generally practiced in intensive growing systems [3]. Considering the fact that the common carp is a cheap fish in Romania (4–5 euro/kg), and the fact that feeding is about half of the total cost of production, it is very important that the feed ingredients be available at a low price. During the COVID-19 pandemic, the price of cereals has risen because of the high demand globally and increased production cost (energy and fuel). The FAO Cereal Price Index reached 145.2 points in August 2022 compared to 2014–2016 [4]. In this context, to ensure the resilience of traditional aquaculture, farmers must turn to cheaper ingredients available in large quantities. At the same time, researchers are making efforts to replace fish meal, an essential ingredient due to its biochemical composition and high digestibility, with ingredients such as plant and animal by-products, micro algae, insects, or single-cell proteins, but these new ingredients must meet numerous criteria, such as adequate nutritional composition, be palatable for fish, available at a low price, etc. [5].

Distiller’s dried grains with solubles (DDGS) are a coproduct of the grain-based ethanol industry. DDGS is readily available at a competitive price on a per unit protein basis compared with other protein sources [6,7]. DDGS is considered to be a competitive source of protein and energy on the world feed market, with a moderately high protein content typically around 30%; however, it can vary between 25% and 45% depending on the source of origin [8]. DDGS is used successfully in the feeding of pigs, sheep, cattle, rabbits etc. [7,9,10]. It has also been tested on various species of fish such as tilapia, trout, turbot, sea bream, etc., with results that vary depending on the type of nutrition of the species (carnivore, omnivore, or vegetarian). For example, in juvenile gilthead seabream [11] and trout [5], DDGS inclusion in the diet did not affect the growth parameters, while in turbot they were affected [12]. In European perch (*Dicentrarchus labrax*) DDGS can be used in combination with phytase with very good results on growth parameters [13]. In hybrid tilapia, DDGS can be used up to 50% if supplemented with lysine [14]. The use of DDGS in the diet of omnivorous fish is all the more attractive as its protein content is close to the nutritional needs of the species. Moreover, as demonstrated in the case of *Piaractus mesopotamicus* juveniles, the inclusion of DDGS in the fish diet has a stimulating effect on the physiological state by improving oxidative status and the health of the intestines, having a prebiotic role [15]. Finding new alternative ingredients to those currently used to feed different species of fish is a topical challenge of applied research in aquaculture [4]. An important advantage of using DDGS in fish feed as an alternative source of protein and nutrients is that, unlike soybean or rapeseed meals, it does not contain anti-nutritional factors [16]. Other advantages of using DDGS in carp feeding would be its relatively low price, high availability, and its fairly balanced chemical composition relative to the nutritional requirements of cyprinids. However, the use of DDGS also has some disadvantages, such as its high content of nondigestible starch polysaccharides [11], which can be reduced by fractionation [17].

In animals, intestinal microbiota play a crucial role in digestion, influencing feeding behavior and the absorption of nutrients. Furthermore, the intestinal microbiota have an important role in the protection against diseases. These functions are assured through bidirectional gut-brain communication (gut–brain axis), which is mediated by hormonal, immune, and neural signals [18]. According to He et al. (2013) [19], DDGS can modulate the gut microbiota of fish in a host-specific way. Despite the importance of the gut microbiome of fish to their host, little is known about the gut microbiome [20]. The gut is colonized by a variety of autochthonous (true) and allochthonous (transient) microorganisms that influence the health of the host, which in turn is directly influenced by the genotype of the fish [20,21] and the host habitat [22], but also captivity, the type of feeding and the quality of the environment in which the aquatic species develop [23,24]. Moreover, knowing the composition of the intestinal microbiota in fish is important because, under stressful conditions, certain species that are normally found in water (*Pseudomonas fluorescens*, *Aeromonas hydrophila*, *Edwardsiella tarda*, *Vibrio* sp., and *Myxobacteria*) can cause epizootic diseases [25].

In this context, the aim of this study was to test the effect of replacing sunflower meal with DDGS in the common carp diet on the main growth indicators, blood biochemical profile, flesh quality, and oxidative status. Furthermore, considering the complexity of the microbiota and the interrelationships between factors that can influence gut microbial populations, four microbiological indicators were selected that, due to their variation, may provide relevant conclusions for our study.

## 2. Materials and Methods

### 2.1. Fish Farming and Management

The biological material used in this study consisted of one summer old common carp (*Cyprinus carpio* L.) fished from the pond and transported to the Recirculating Aquaculture System (RAS) system where they were allowed to acclimatize for two weeks. After this period, the fish were left without food for 24 h, weighed, and those with a uniform initial weight (86 g) were selected. The animals were assigned into three diet groups (D0, D1, and D2) and three replicates (20 fish/replicate). The RAS is equipped with fiberglass growth tanks (0.75 m^3^/tank), mechanical drum type filter, UV filter, biological filter, pump supply system, and sensors for monitoring the water physico-chemical parameters. Fluorescent tubes were used to supplement the natural light for 10 h/day. The experiment lasted seven weeks. Feeding was performed manually three times a day (at 8, 12 and 15 pm). The feeding ratio was determined according to the weight of the fish and the water temperature. The temperature and content of dissolved oxygen were measured with a Hach HQ30d (Hach Company, Loveland, CO, USA) portable oxygen meter, pH, and conductivity with a Hach HQ11d (Hach Company) portable multiparameter, while ammonia, nitrites, nitrates, and phosphates were assessed with a Hanna Iris HI801 Spectrophotometer and Hanna reagent kits (Hanna Instruments, Salaj, Romania). The water parameters are presented in Table 1.

### 2.2. Experimental Diets

To test the effect of DDGS on common carp, three experimental diets (D0, D1 and D2) were formulated. The first diet, D0, consists of sunflower meal, corn, wheat, and fish meal. D0 represented the control diet because it does not contain DDGS. The following two diets, D1 and D2, contain, in addition to the abovementioned ingredients, DDGS (25% and 35%, respectively). Table 2 shows the ingredients used in the experimental diets. The diets were made by extruding the ingredients, grinding, mixing, and pelleting.

### 2.3. Evaluation of Growth Performance

The following morphometric measurements were performed weekly: body mass (g), total length (cm), standard length (cm), height (cm), maximum thickness (cm), circumference (cm), and head length (cm). Based on the measurements performed, the main growth indices were calculated [27,28].

IBW—initial body weight (g);

FBW—final body weight (g);

WG—weight gain (g) = FBW − IBW;

FCR—feed conversion ratio (g/g) = Feed intake (g)/WG;

RGR—relative growth rate (g/g day^−1^) = WG/days of experiment/IBW;

SGR—specific growth rate (% day^−1^) = (ln FBW − ln IBW)/days of experiment × 100;

PER—protein efficiency ratio = WG/total protein;

CF—condition factor = FBW/body length^3^ × 100;

HSI (hepatosomatic index, %) =100 × [final liver weight (g)/final body weight (g)];

VSI (viscerosomatic index, %) =100 × [final visceral weight (g)/final body weight (g)].

### 2.4. Proximate Composition of Diets and Fish Meat

The proximate composition of feed and meat was performed using a DA 7250 NIR Analyzer, (Perten Instruments, Hagersten, Sweden).

### 2.5. Blood Biochemical Profile

The fish were anesthetized with clove oil (2%), and their blood was sampled via heart puncture. The samples were analyzed with the MNCHIP Pointcare V2 Analyzer [29]. The biochemical parameters were: ALB—albumin, TP—total protein, GLO—globulin, A/G—albumin/globulin, Ca^2+^—calcium, GLU—glucose, BUN—blood urea nitrogen, AMY—amylase, CHOL—cholesterol, ALT—alanine aminotransferase, TBIL—total bilirubin, ALP—alkaline phosphatase, CRE—creatinine, CK—creatine kinase.

### 2.6. Biochemical Parameter Assessment

Shortly after the diet period ended, all fishes were euthanized with a 2% clove oil solution and liver and muscle tissue samples were precisely dissected and collected for oxidative status assessment. The tissue samples were homogenized in an ice-cold potassium phosphate buffer solution 0.1 M, KCl 1.15%, pH 7.4 in a ratio of 1:10 (*w*/*v*). The homogenates were centrifuged (20 min at 3000 rpm and 4 °C) and the supernatants were further used to measure the activities of superoxide dismutase (SOD), catalase (CAT) and glutathione peroxidase (GPX), and to determine the content of reduced glutathione (GSH), malondialdehyde (MDA) and carbonylated proteins, according to the methods described in Capatina et al. (2020) [30]. The SOD, CAT and GPX activities and the levels of GSH, MDA and carbonylated proteins were normalized to the total content of soluble proteins measured by the Bradford method (Bradford, 1976) [31].

### 2.7. Intestinal Microbiota

The microbiological examination consisted of the quantitative determination of the existing microorganisms in the intestinal contents of the carp (total number of aerobic microorganisms, total number of anaerobic microorganisms, the total number of sulphite-reducing Clostridia, and the total number of Enterobacteriaceae) [32].

#### 2.7.1. Determination of the Total Number of Aerobic Microorganisms in the Intestinal Contents

From each intestinal mass sample, 1 g of intestinal content was collected, from which decimal dilutions were made in physiological serum (10^−1^–10^−6^). From the dilutions made, with sterile 1 mL pipettes, after homogenization, 1 mL was distributed in 2 Petri dishes, over which approximately 15 mL of Mueller Hinton Agar (MH, Ecolab) was poured, melted, and cooled to 45 °C so as not to destroy the microbial cells. After solidification, the plates were incubated under aerobic conditions at a temperature of 37 °C. After 48 h, the samples were examined, and the bacterial colonies formed on and in the structure of the medium were counted with the naked eye or with a colony counter. All the dilutions were performed in duplicate; in order to compare the results, the dilution was averaged, and the result was multiplied by the dilution factor. The result was expressed in colony-forming units/g intestinal content.

#### 2.7.2. Determination of the Total Number of Anaerobic Microorganisms in the Intestinal Contents

To determine the number of anaerobic germs, the same decimal dilutions obtained initially were used, from which 1 mL of suspension was distributed in sterile Petri plates on top of which Columbia Agar Base with 5% Defibrinated Horse Blood was added, this being a medium of culture adapted for the development of anaerobic bacteria. Incubation was carried out under anaerobic conditions, using an anaerobic jar in which anaerobic GenBag systems (bioMérieux France) were inserted.

#### 2.7.3. Determination of the Total Number of Sulphite-Reducing Clostridia in the Intestinal Contents

To determine the number of sulfite-reducing Clostridia, the same working method applied to the anaerobic microorganisms was used, with the exception of the culture medium used, namely SPS Agar (Sulfite-Polymyxin B-Sulfadiazine).

#### 2.7.4. Determination of the Total Number of Enterobacteriaceae in Intestinal Contents

The work technique was similar to the previous one, though the culture medium used was Bromocresol purple lactose Agar (BCP).

### 2.8. Statistical Analysis

The growth performance, flesh quality, blood biochemical profile and intestinal microflora data were statistically processed by analysis of variance (ANOVA) followed by Tukey Test (*p* < 0.05) using the SPSS software version 21 (IBM Corp, Armonk, NY, USA) [33]. The results were reported as means ± standard error of men (S.E.M.). The biochemical results were analyzed by two-way ANOVA followed by Tukey’s multiple comparisons tests using GraphPad Prism software v9.3.1 (La Jolla, CA, USA). The significant differences were considered when *p* < 0.05 and the values were expressed as means ± S.E.M.

## 3. Results

### 3.1. Diet Composition

The proximate composition of diets is presented in Table 3. The ingredients used in this study are commonly used in common carp feeding in Romania, with the exception of DDGS. The protein content was significantly (*p* ˂ 0.05) lower in D2, decreasing by 5% compared to D0, while the fat content was significantly (*p* ˂ 0.05) higher in D2 and D1, increasing by 59% and 50%, respectively, compared to D0. A greater variation in the composition of the diet was recorded at D2, thus, the content of fiber, starch and calcium decreased significantly (*p* ˂ 0.05) compared to D0 by 13%, 7%, and 11%, respectively, while the content of sugar and phosphorus increased significantly (*p* ˂ 0.05) compared to D0 by 165% and 200%, respectively.

### 3.2. Fish Growth Performance

The growth parameters of common carp are shown in Table 4. The variations were small and statistically insignificant (*p* > 0.05) for all parameters. However, it must be specified that the feed conversion factor, one of the most important indices showing the efficiency of feed use, decreased by 39% in D1 and by 27% in D2 compared to D0.

### 3.3. Flesh Proximate Composition

The proximate composition of meat is presented in Table 5 The variations were small, thus, the inclusion of DDGS in the diet did not affect the quality of the meat. The fat content was lower in D2 by 23% and higher in D1 by 14% compared to D0, however, the differences were not significant (*p* > 0.05). The only significant differences compared to D0 were recorded for the salt and ash parameters, with a 53% decrease in salt content at D1 and a 32% increase in ash content at D2.

### 3.4. Blood Biochemical Profile

Table 6 presents the blood biochemical profile of common carp. The variations are small and statistically insignificant (*p* > 0.05), which demonstrates that the inclusion of DDGS in the diet does not affect the blood parameters in carp.

### 3.5. Oxidative Status

**Figure 1:**
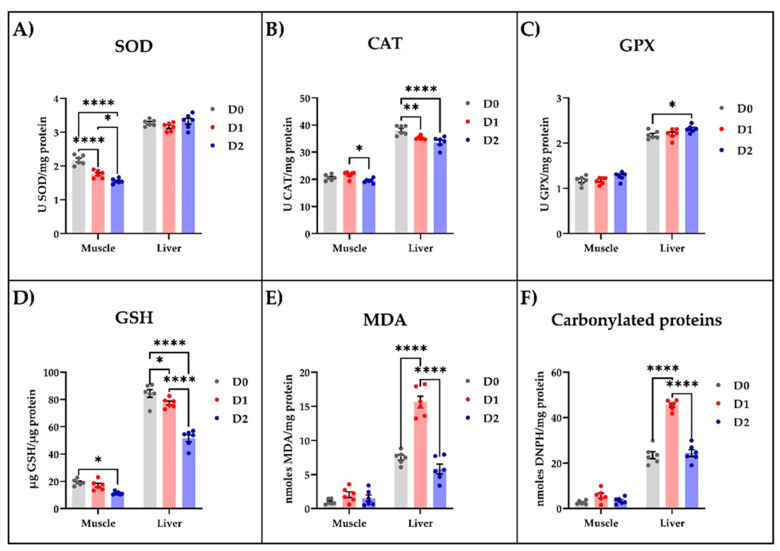
The influence of different types of diet on the enzymatic and non-enzymatic parameters of oxidative stress measured in muscle and liver tissue of carp. The enzymatic parameters consisted of measuring the (**A**) SOD, (**B**) CAT, and (**C**) GPX specific activities, while the non-enzymatic parameters consisted of estimating the levels of (**D**) GSH, (**E**) MDA, and (**F**) carbonylated proteins. The values are expressed as means ± S.E.M. (*n* = 6). Two-way ANOVA analysis revealed overall significant differences between the experimental groups in (**A**) F (2,30) = 17.07, *p* < 0.0001; (**B**) F (2,30) = 13.31, *p* < 0.0001; (**C**) F (2,30) = 5.962, *p* < 0.01; (**D**) F (2,30), *p* < 0.0001; (**E**) F (2,30) = 51.34, *p* < 0.0001; and (**F**) F (2,30) = 80.32, *p* < 0.0001. For Tukey post hoc analysis, **** *p* < 0.0001, ** *p* < 0.01, * *p* < 0.05.

Following the biochemical analysis of muscle and liver tissue samples, we noticed that the DDGS-based diet influenced the oxidative status (Figure 1). In the muscle tissue, this diet significantly reduced, in a dose-dependent manner, the specific activity of SOD (*p* < 0.0001, Figure 1A), while the CAT (Figure 1B) and GPX (Figure 1C) specific activities were left unaffected. However, the DDGS-fed carp exhibited a decrease only in CAT specific activity (Figure 1B) in the liver tissue compared to the fish fed with standard food. Both DDGS-based diets, especially the one with the increased content of DDGS, significantly lowered the GSH level in muscle and liver compared to the fish fed with standard food (Figure 1D). Moreover, low levels of MDA (Figure 1E) and carbonylated proteins (Figure 1F) were detected in the muscle tissue of all animals whereas, in the liver, only the diet richer in DDGS was able to significantly (*p* < 0.0001) reduce these parameters compared with the control group.

### 3.6. Intestinal Microbiota

The intestinal microbiological analysis of common carp was represented by: the total number of aerobic bacteria (TNA), total number of anaerobic bacteria (TNAN), total number of sulfite-reducing clostridia (TNC), and total number of Enterobacteriaceae (TNE). It was found that the microbial density varied depending on the diet used (Supplementary Table S2). The mean TNA (cfu/g) was 224.2 × 10^4^ in D0, 366 × 10^4^ in D1, and 201.6 × 10^5^ in D2. The mean TNAN (cfu/g) was 176.8 × 10^1^ in D0, 152 × 10^1^ in D1, and 288 × 10^1^. The mean TNC (cfu/g) was 402.6 in D0, 750.8 in D1, and 739 in D2. The mean TNE (cfu/g) was 105.94 × 10^4^ in D0, 621.68 × 10^4^ in D1, and 120.648 × 10^5^ in D2.

**Figure 2:**
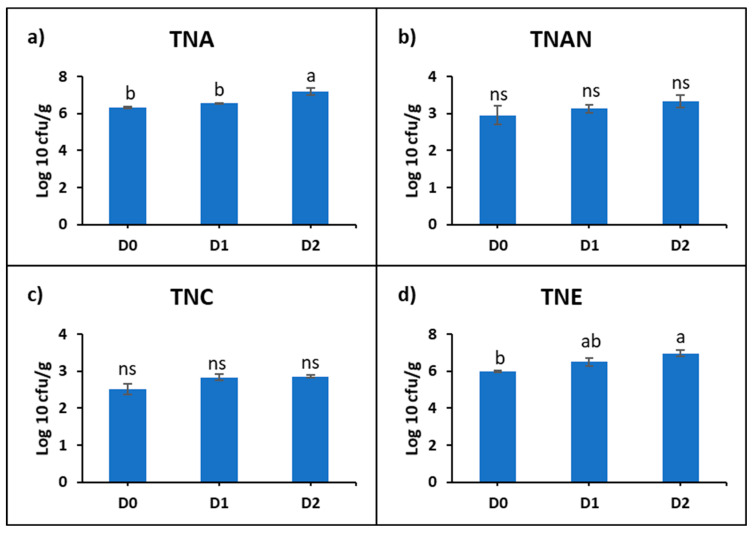
Representation of logarithmic means of: (**a**) total number of aerobic bacteria (TNA), (**b**) total number of anaerobic bacteria (TNAN), (**c**) total number of sulfite-reducing clostridia (TNC), (**d**) total number of Enterobacteriaceae (TNE) in the intestine of common carp fed with experimental diets with DDGS. Lowercase letters represent statistically significant differences according to Tukey’s test (*p* ˂ 0.05), ns—not significant.

The statistical analysis of the logarithmic values showed a progressive increase in the microbial density correlated with the concentration of DDGS in the diet. Statistically significant differences were recorded at D2 compared to D0, with increases of 13% at TNA and 15% at TNE (Figure 2).

## 4. Discussion

The aim of this study was to identify the optimal dose of DDGS to replace sunflower meal in the common carp diet and to ensure normal growth without affecting the growth and physiological parameters of the species. To achieve this goal, the growth parameters, meat quality, blood biochemical profile, oxidative status and intestinal flora were investigated. An important issue related to the sustainability of feed formulation and ingredient selection is the need to ensure the nutritional requirements of the species for optimal and healthy growth at the desired density and for the cultivation system [34]. The DDGS used in this study has a relatively high protein content of 27.56%, while the complete composition was: oil 6.7%, fiber 10.13%, ash 3.6%, ADF 6.6%, NDF 38.3%, starch 1.57%, S 0.47%, P 0.7% (Supplementary Table S1). The content of nutrients in DDGS varies depending on the study, the producing factories, or even depending on the batch within the same producer; therefore, it is recommended to analyze the composition of each new batch that is to be used as an ingredient in the feed production [35]. Olukosi and Adebiyi (2013) [36] analyzed the data of several studies and determined that the chemical composition of maize DDGS varied among sources, having a coefficient of variation (%) of 8.5 for crude protein (347–233 g/kg), 15.1 for crude fiber (113–62 g/kg), 15.7 for neutral fiber detergent (510–277 g/kg), 24.2 for acid fiber detergent (185–86 g/kg), 22.0 for ether extract (177–32 g/kg), 13.6 for ash (59–31 g/kg), 8.8 for total phosphorus (9.8–6.9 g/kg), and 53.5 for calcium (0.8–0.2 g/kg). Sunflower meal in this study had a high protein content of 40.6% and a low fat content of 1.76%, so replacing it with DDGS led to a decrease in the protein content of D2 by 5% and an increase in fat content of 59% compared to the control version D0. However, the inclusion of DDGS in the diet did not cause statistically significant changes in the growth parameters (FCR, WG, SGR, RGR, PER, CF, HSI, VSI), which highlights the opportunity of inclusion of DDGS in the common carp diet. Better results were recorded by Révész et al., 2019 [37], who established that the administration of up to 40% DDGS in the diet of common carp with an initial weight of 63 g causes a significant increase in the parameters FBW, WG, FCR, PER, PPV. A possible explanation for the high percentage of DDGS that can be included in the diet of omnivorous fish, i.e., common carp, can be the protein content of DDGS, which is close to the requirements of these species (30–35%) [38]. However, as stated above, the influence of DDGS on growth parameters may vary depending on its source, as shown by Abouel Azm et al., 2022 [16], who reported better results in the growth parameters FBW, SGR, and FE in grass carp (*Ctenopharyngodon idellus*) fed with DDGS imported from the USA compared to native DDGS (China). Although the FCR decreased in the present study by 30% in the DDGS diets, the differences were not significant. By comparison, Révész et al., 2019 [37] recorded a significant decrease in FCR, from 2.08 to 1.81, by including 40% DDGS in the common carp diet. Similarly, Sándor et al., 2021 [39] obtained a significant reduction in FCR in carp with an initial weight of 363 g fed a 40% DDGS diet from 1.78 to 1.56 g/g.

Regarding the influence of DDGS on flesh composition, the variations were small in the present study, especially in the case of fat and protein content. The flesh composition is important, especially from the nutritional perspective when used for human consumption. For example, fat content is one of the most important quality indicators of fish meat, this being influenced by diet and technological factors [40]. At the European level, there are countries where the sale of carp meat is accepted only if the fat content is less than 10%, because a higher content negatively affects the taste of the meat [41]. The fat content in this study decreased in the D2 group, but the differences were not significant. Sándor et al. (2021) [39] indicated an increase in fat content in market size common carp from 5.92% (control) to 6.16% (experimental variant with 40% DDGS) and a decrease in protein content from 16.81% to 16.79%, however, the variations were insignificant. In the same study, there were also no differences in the water and ash content, the only significant variation being recorded in the total n-6, which increased from 7.7 to 9.98. Similarly, the use of DDGS in the diet of different species (catfish and rainbow trout) did not produce significant changes in meat composition [42,43].

In this study, the variations on the blood biochemical profile were small and the differences statistically insignificant, which proves that DDGS does not affect the blood parameters. Our results are in agreement with those obtained by Révész et al., 2019 [37], who showed that the inclusion of DDGS in the carp diet of up to 40% did not produced changes in the blood parameters TC, TG, ALT, AST, AP, GGT, amylase, and lipase. Similarly, in grass carp (*Ctenopharyngodon idellus*), the inclusion of 20% and 30% DDGS from two sources (China and USA) in the diet did influence the following blood parameters: ALAT, ASAT, TP, glucose, triglyceride, and total cholesterol. Moreover, the same study reported that there were no histological changes in hepatocyte structure between the experimental groups [16]. However, it is known that blood parameters can vary greatly depending on the fish species and age, the environmental conditions, and the treatments used. For example, the inclusion of 4% wheat juice in the common carp diet caused an increase in total protein, albumin, globulin, and Ca suggesting an immunological role of this ingredient [29]. The same parameters also increased in carp exposed to infection with *Aeromonas hydrophilla* fed with a diet supplemented with *Gingko biloba* leaf extract [44]. Although the administration in the diet of some vegetable preparations, such as black seed in rohu, can determine a nephroprotective effect by decreasing creatinine and urea levels, and a hepatoprotective effect by decreasing the activity of ALP, AST, and ALT enzymes [45], the fact that in the present study these parameters varied insignificantly demonstrate that animal welfare is not affected by DDGS.

Regarding oxidative status, in this study, DDGS produced a dose-dependent decrease in the activity of SOD in muscle tissue, CAT in liver tissue, and GSH in both investigated tissues. Moreover, MDA and carbonylated proteins were reduced in the liver of those on the DDGS diet. The need to increase production densities in aquaculture to ensure profitability may affect the well-being of animals through the occurrence of oxidative stress at the physiological level [46]. SOD, CAT, GPX and GPH enzymes are part of the response mechanism to oxidative stress through the conversion of superoxide radicals and hydrogen peroxide to O_2_ and H_2_O, while MDA and carbonylated proteins are markers for oxidative stress [47]. One way to reduce oxidative stress caused by high cultivation densities or environmental conditions is the use of diets with antioxidant potential. For this reason, research aimed at identifying ingredients with antioxidant potential are topical [48,49,50,51,52]. In general, an increased activity of antioxidant enzymes signals the presence of stress [53,54]. As in the case of the parameters discussed above, the effect of the diet on oxidative status in fish can vary, both depending on the ingredients tested and on the species. For example, the dietary replacement of fishmeal by 10, 17.5, or 25% DDGS in turbot (*Scophthalmus maximus)* juveniles did not affect the specific activity of GPX, GR, CAT, and SOD and lipid peroxidation values in the liver and posterior intestine [12]. Similarly, replacing soybean meal with 0, 100, 200, 300 and 400 g of DDGS/kg for pacu (*Piaractus mesopotamicus*) juveniles, which is an omnivorous characin, did not produce significant variations in glutathione peroxidase, GR, and SOD activities in intestine tissue, while a quadratic effect of dietary DDGS on CAT activity was obtained, and G6PDH activities and LPO linearly decreased with dietary DDGS level [15]. An increase in CAT and peroxidase activities was observed in common carp after supplementing the diet with polyphenols, determined as a result of triggering the antioxidant mechanism caused by polyphenols [55], while Dumitru et al., 2018 [47], reported an increase in SOD, CAT and GPX in muscle and liver in common carp after the inclusion of 4% wheatgrass juice in the diet.

In this study, the development of aerobic and anaerobic bacterial populations correlates with the concentration of DDGS used as an ingredient for common carp feed, indicating that the chemical and nutritional composition of the feed was conducive to the proliferation of the gut microbiome. The high protein (26%) and lipid (7%) content of DDGS had a statistically significant effect on the aerobic bacterial population (13%) and an insignificant effect on the anaerobic microbial population. In a review, Riaz Rajoka et al., 2021 [56], highlighted the positive impact of diets with plant proteins on the gut microbiome by increasing the composition of some species (*Enterococcus*, *Bifidobacterium*, and *Lactobacillus*) and by increasing the production of short chain fatty acids with anti-inflammatory properties. The gut microbiota of fish changes with the developmental stages of the organism [57], and their gut is capable of harboring an aerobic and anaerobic microbial population of up to 1011 bacteria per gram of gut content [58]. Moreover, the microbiota composition may change with temperature, feeding behavior, and host specific characteristics [44]. Al-Harbi and Uddin (2008) [26], reported total viable bacterial counts in the common carp intestine of 8.7 ×10^9^ to 5.4 ×10^10^ cfu/g, which was higher compared to the current study, and with the following composition *Aeromonas hydrophila*, *Shewanella putrefaciens*, *Vibrio cholerae*, *Staphylococcus sp*., and *Vibrio vulnificus*. Ofek et al., 2021 [59] investigated the microbiota composition of five fish species (hybrid striped bass *Moron saxatilis* × *Moron chrysops*, European bass *Dicentrarchus labrax*, red drum *Sciaenops ocellatus*, hybrid tilapia *Oreochromis aureus* × *Oreochromis niloticus*, flathead grey mullet *Mugil cephalus*, and common carp *Cyprinus carpio*) and found that the composition varied based on the fish species, sampling seasons, and between fish sizes, and that the most abundant genus in common carp were *Cetobacterium* and *Aeromonas*.

On the other hand, sulfite-reducing clostridia (bacteria that have the property of reducing sulfites to sulfides) also belong to the group of anaerobic bacteria. Clostridia are much more abundant in the gut of freshwater fish than in that of saltwater fish, especially in fish that feed on plants [22]. These microorganisms can form endospores that allow the bacteria to survive in almost any habitat, whether on land or in water, waiting for favorable growth conditions [60]. Since only anaerobes with spores of intestinal origin can reduce sulfites [61], we were able to identify this group of microorganisms as a microbiological indicator. In our study, the load of sulfite-reducing clostridia was much lower (402.6–739 cfu/g) than in other studies (102–104 cfu/g) conducted on healthy fish and under natural growth conditions [61]. Spores of sulphite-reducing Clostridia are ecologically relevant as they are an important indicator of fecal contamination of water and resist in this environment much longer than *Salmonella*. When organic pollution increases, proteolysis and ammonification of the sulphite-reducing clostridia can be activated, leading to the formation of large amounts of hydrogen sulphide and ammonia. Newly added pollutant compounds cause real changes in the functioning of all oceanic biocenoses and disturb the natural harmony of this environment [61].

A statistically significant increase (15%) was also found for the indicator Enterobacteriaceae (TNE). These aerobic (facultative anaerobic) Gram-negative microorganisms are part of the normal microbiota of fish [62]. In a recent study, the authors correlated the presence of enterobacteria with reduced digestibility in fish fed alternating diets based on animal and vegetable protein meal [63]. It was concluded that enterobacteria altered the symbiotic balance in the gut, allowing colonization by opportunistic bacteria and leading to a reduction in nutrient digestibility. Our studies have shown that increasing the number of *Enterobacteriaceae* microorganisms had no negative effect on the metabolism and digestibility of proteins in the feed administered and did not affect the development and health of fish fed DDGS. The results of our study support the findings of previous research [59] showing that fish feed can influence the gut microbiota of healthy fish from aquaculture.

## 5. Conclusions

The results of this study showed that at a concentration of 35% DDGS (D2) in the diet, growth parameters and flesh quality were not affected. More specifically, although the FCR decreased from 3.94 D0, to 2.39 D1, and 2.90 D2, the differences were not statistically significant. Regarding flesh quality, the fat content decreased in the D2 variant, from 4.43% D0 to 3.38% D2, but the differences were not significant, and increased significantly in D1, reaching 5.06%. From a physiological point of view, the blood biochemical profile was not affected, while the oxidative status improved, and the intestinal microbiota were positively affected. This study demonstrates that adding 35% DDGS and reducing sunflower meal from 35% to 5% in the diet does not affect carp growth and development. These results can be used to formulate practical feed diets with DDGS for common carp.

## Figures and Tables

**Table 1:** Physico-chemical parameters of water.

Parameters	Measure Units	Values	Reference Values [26]
Temperature	°C	22.00	max. 28.00
pH	Units	8.30	6.5–8.50
Dissolved oxygen (DO)	mg/L	6.60	min. 5.00
$\mathrm{Nitrate}\;({\mathrm{NO}}_{3^{-}}$)	mg/L	below detection limit	30.00
$\mathrm{Nitrite}\;({\mathrm{NO}}_{2^{-}}$)	mg/L	0.16	3.00
$\mathrm{Ammoniac}\;({\mathrm{NH}}_{3}^{+}$)	mg/L	0.09	0.30
$\mathrm{Ammonia}\;({\mathrm{NH}}_{4}^{+}$)	mg/L	0.10	3.00
$\mathrm{Phosphate}\;({\mathrm{PO}}_{4^{3-}}$)	mg/L	0.01	0.10

**Table 2:** The composition of ingredients used in the experimental diets with DDGS for common carp.

Ingredients/Experimental Variant	D0	D1	D2
Sunflower meal	35%	20%	5%
Corn	25%	20%	20%
Wheat	20%	15%	20%
Fish meal	20%	20%	20%
DDGS	0%	25%	35%

**Table 3:** Proximate composition of diets for common carp containing DDGS.

Parameters	D0	D1	D2
Moisture %	9.12 ± 0.14 b	9.14 ± 0.1 b	10.51 ± 0.12 a
Protein %	26.53 ± 0.31 a	26.45 ± 0.3 a	24.97 ± 0.12 b
Fat %	3 ± 0.13 c	4.51 ± 0.09 b	4.78 ± 0.1 a
Fiber %	6.39 ± 0.17 a	6.29 ± 0.11 a	5.5 ± 0.07 b
Amidon %	44.02 ± 0.46 a	41.03 ± 0.12 b	40.88 ± 0.25 b
Ash %	7.6 ± 0.17 b	7.85 ± 0.15 a	7.57 ± 0.12 b
Sugar %	1.27 ± 0.21 c	2.34 ± 0.12 b	3.37 ± 0.15 a
Calcium %	1.78 ± 0.18 a	1.61 ± 0.08 ab	1.58 ± 0.07 b
Phosphorus %	0.2 ± 0.06 c	0.41 ± 0.03 b	0.6 ± 0.04 a

Lowercase letters represent statistically significant differences according to Tukey’s test (*p* ˂ 0.05).

**Table 4:** Growth parameters of common carp fed with experimental diets with DDGS.

Parameter	D0	D1	D2	ANOVA (*p*-Value)
FCR	3.98 ± 1.71	2.39 ± 0.91	2.90 ± 1.53	0.72
WG	1381.00 ± 296.44	1376.72 ± 359.24	1367.22 ± 330.81	1.00
SGR	0.85 ± 0.05	0.83 ± 0.05	0.83 ± 0.09	0.96
RGR	23.84 ± 5.12	24.72 ± 6.45	24.47 ± 5.92	0.99
PER	1.80 ± 0.51	0.79 ± 0.15	0.82 ± 0.23	0.08
CF	1.78 ± 0.07	1.75 ± 0.09	1.81 ± 0.10	0.66
VSI	10.55 ± 0.40	8.97 ± 0.42	10.06 ± 0.62	0.20
HSI	1.12 ± 0.08	0.83 ± 0.11	0.95 ± 0.09	0.24
S (%)	100.00	100.00	100.00	1.00

**Table 5:** Proximate composition of common carp meat fed with experimental diets with DDGS.

Diets	D0	D1	D2	AVOVA (*p*-Value)
Fat As is %	4.43 ± 0.35 ab	5.06 ± 0.34 a	3.38 ± 0.38 b	0.01
Moisture %	73.40 ± 0.26	72.51 ± 0.37	73.36 ± 0.39	0.14
Protein As is %	17.00 ± 0.13	16.18 ± 0.24	16.87 ± 0.30	0.04
Collagen As is %	0.68 ± 0.12	1.02 ± 0.15	0.89 ± 0.10	0.16
Salt As is %	1.12 ± 0.17 a	0.52 ± 0.15 b	0.68 ± 0.12 ab	0.02
Ash As is %	1.60 ± 0.13 b	1.92 ± 0.1 ab	2.12 ± 0.12 a	0.01

Lowercase letters represent statistically significant differences according to Tukey’s test (*p* ˂ 0.05).

**Table 6:** Blood biochemical profiles of common carp fed with experimental diets with DDGS.

Parameters	D0	D1	D2	ANOVA (*p*-Value)
ALB (g/dL)	1.70 ± 0.03	1.77 ± 0.07	1.70 ± 0.03	0.81
TP (g/dL)	3.47 ± 0.12	3.43 ± 0.3	3.60 ± 0.06	0.93
GLO (g/dL)	1.77 ± 0.08	1.67 ± 0.24	1.87 ± 38.34	0.42
A/G	0.97 ± 0.04	1.20 ± 0.17	0.90 ± 0.03	0.29
Ca (mg/dL)	10.70 ± 0.35	9.70 ± 0.47	9.73 ± 0.15	0.11
GLU (mg/dL)	103.33 ± 7.78	157.33 ± 19.37	134.00 ± 6.57	0.39
BUN (mg/dL)	6.83 ± 0.17	8.26 ± 0.43	6.13 ± 0.41	0.51
P (mg/dL)	9.46 ± 0.68	7.26 ± 0.78	8.36 ± 0.18	0.74
CHOL (mg/dL)	223.67 ± 5.27	273.67 ± 33.98	219.67 ± 4.03	0.80
ALT (U/L)	318.33 ± 103.25	335.33 ± 54.49	213.67 ± 13.57	0.50
TBIL (mg/dL)	0.22 ± 0.02	0.22 ± 0.02	0.19 ± 0.01	0.46
ALP (U/L)	129.67 ± 31.58	135.00 ± 37.35	66.67 ± 4.68	0.58
CRE (mg/dL)	0.55 ± 0.07	0.74 ± 0.10	0.71 ± 0.09	0.66
BUN/CRE	13.67 ± 2.22	14.00 ± 3.18	10.00 ± 1.76	0.77

## Data Availability

Not applicable.

## Supplementary Materials

The following supporting information can be downloaded at: https://www.mdpi.com/article/10.3390/life12101609/s1. Table S1: Proximate composition of ingredients used for common carp diet; Table S2: Determination of aerobic and anaerobic bacterial density cfu/g intestinal content of common carp.
