# Supplementary material for: Evaluation of DDGS as a Low-Cost Feed Ingredient for Common Carp (Cyprinus carpio Linneus) Cultivated in a Semi-Intensive System
Source: Life (Basel). 2022 Oct 14;12(10):1609. doi: 10.3390/life12101609 (PMC9604809; doi:10.3390/life12101609)
Supplement: Supplementary file 1 [file life-12-01609-s001.zip › life-1952161-supplementary.pdf]

Table S1. Proximate composition of ingredients used for common carp diet

Parameter	DDGS	Corn	Wheat	Fish meal	Sunflower meal
Humidity %	11.5	13.63	14.02	9.50	9.93
Protein %	27.56	7.54	8.07	39.9	40.6
Oil %	6.75	3.10			
Fiber %	10.13				22.6
Ash %	3.6			24.3	7.63
ADF %	16.6				
NDF %	38.3		26.80		
Starch %	1.57	66.66	68.8		
S %	0.47				
P %	0.7				
Fat %				8.9	1.76
N total mg/100g				242	
Histamine ppm				12100	
FFA				77.88	
Acidity				50.32	

Table S2. Determination of aerobic and anaerobic bacterial density cfu / g intestinal content of common carp

Parameter / treatment	Sample 1	Sample 2	Sample 3	Sample 4	Sample 5	Mean
TNA D0	2360000	1980000	1680000	2210000	2980000	2242000
TNA D1	3320000	4210000	3020000	3820000	3930000	3660000
TNA D2	23900000	33100000	28900000	11200000	3700000	20160000
TNAN D0	1200	6000	320	220	1100	1768
TNAN D1	700	1200	1100	1700	2900	1520
TNAN D2	1100	2900	3300	700	6400	2880
TNC D0	620	600	200	110	483	402.6
TNC D1	522	394	647	891	1300	750.8
TNC D2	720	800	722	520	933	739
TNE D0	1414000	970000	705000	1016000	1192000	1059400
TNE D1	1762000	22980000	1812000	2330000	2200000	6216800
TNE D2	13623000	19529000	17975000	6977000	2220000	12064800

TNA - total number of aerobic microorganisms
TNAN - total number of anaerobic microorganisms
TNC - total number of sulfite-reducing clostridia
TNE - total number of Enterobacteriaceae
CFU/g= colony-forming unit per gram
